# Effects of 21-day vitamin D supplementation on sports performance and exercise-induced inflammation: a randomized double-blind trial

**DOI:** 10.3389/fphys.2026.1867500

**Published:** 2026-09-04

**Authors:** Katarzyna Grześkiewicz, Paulina Brzezińska, Andżelika Borkowska, Andrzej Kochanowicz, Błażej Stankiewicz, Elżbieta Piskorska, Tomasz Waldziński, Pavol Bartík, Jędrzej Antosiewicz, Jan Mieszkowski

**Affiliations:** 1 Department of Gymnastics and Dance, Gdańsk University of Physical Education and Sport, Gdańsk, Poland; 2 Division of Clinical Physiotherapy, Faculty of Physical Education, Gdansk University of Physical Education and Sport, Gdańsk, Poland; 3 Department of Bioenergetics and Physiology of Exercise, Medical University of Gdańsk, Gdańsk, Poland; 4 Department of Biomedical Basis of Physical Education, Kazimierz Wielki University, Institute of Physical Education, Bydgoszcz, Poland; 5 Department of Pathobiochemistry and Clinical Chemistry, Nicolaus Copernicus University Collegium Medicum, Bydgoszcz, Poland; 6 Faculty of Health Sciences, University of Lomza, Łomża, Poland; 7 Faculty of Sports Science and Health, Matej Bel University, Banská Bystrica, Slovakia

**Keywords:** 25(OH)D, IL-1 beta, IL-10, IL-6, inflammation, vitamin D

## Abstract

**Objectives:**

While an increasing number of studies highlight the significance of vitamin D supplementation for athletic performance, the effects of short-term high-dose vitamin D_3_ supplementation on exercise-induced inflammation remain unclear. This study aimed to evaluate the effects of 21 days of vitamin D_3_ supplementation (10,000 IU/day) on inflammatory biomarkers and maximal anaerobic performance in physically active men performing the 2 × Wingate Anaerobic Test (2xWAnT).

**Methods:**

Thirty physically active men were randomly assigned in a double-blind manner to a vitamin D_3_ group (10,000 IU/day in vegetable oil; n = 15; aged 24.21 ± 4.35) or a placebo group (vegetable oil; n = 15; aged 23.15 ± 3.44). During the supplementation period, one participant from the vitamin D_3_ group and two from the placebo group were excluded because of non-compliance, leaving 27 participants for the final analyses (vitamin D_3_, n = 14; placebo, n = 13). Venous blood samples were collected at rest, immediately after, 3 h after, and 24 h after the 2xWAnT, before and after the 21-day intervention.

**Results:**

Resting 25(OH)D increased by +55.7% after supplementation in the vitamin D_3_ group. Wingate outcomes showed no differential effect of vitamin D_3_ versus placebo on anaerobic performance. At rest, vitamin D_3_ supplementation was associated with lower IL-1β and IL-6 concentrations and higher IL-10 concentrations. After the 2xWAnT, the vitamin D_3_ group showed an attenuated IL-1β response, lower IL-1RA concentrations during the post-exercise period, and higher IL-10 concentrations across the post-exercise period. In contrast, despite the pronounced exercise-induced increase in IL-6, the post-exercise time course of this biomarker did not differ between groups.

**Conclusions:**

Twenty-one days of vitamin D_3_ supplementation increased serum 25(OH)D concentrations but did not improve anaerobic performance. Supplementation was associated with changes in selected resting and post-WAnT inflammatory biomarkers, suggesting modulation of the biochemical inflammatory response to repeated supramaximal anaerobic exercise. Functional recovery outcomes were not assessed.

## Introduction

An excessive inflammatory response to strenuous exercise is increasingly recognized as a factor that may impair athletic performance, recovery, and overall athlete health (Mieszkowski et al., 2021a; Stankiewicz et al., 2023). Proinflammatory cytokines may disrupt skeletal muscle metabolism, alter insulin signaling, and promote central nervous system inflammation, which in turn may contribute to disturbances in motor control and coordination (McFadden et al., 2022). Accordingly, there is growing interest in interventions that may attenuate exercise-induced inflammatory stress in physically active individuals (Nemkov et al., 2025).

Recent studies have highlighted the immunomodulatory and anti-inflammatory properties of vitamin D. Vitamin D has been shown to reduce the synthesis of proinflammatory cytokines, including interleukin-6 (IL-6) and tumor necrosis factor-alpha (TNF-α), in monocytes. In macrophages, the biologically active form 1,25(OH)_2_D_3_ may attenuate inflammatory signaling by upregulating inhibitors of nuclear factor kappa B (NF-κB) (Jablonski et al., 2011). These effects are biologically plausible because vitamin D acts through the vitamin D receptor (VDR), which is expressed in multiple tissues relevant to exercise adaptation, including skeletal muscle and immune cells, and regulates the expression of numerous genes involved in inflammatory control (Matilainen et al., 2010).

Vitamin D supplementation has also been linked to favorable adaptations in the context of exercise training (Mieszkowski et al., 2021b). In animal models, supplementation has been shown to attenuate inflammation induced by high-intensity exercise (Choi et al., 2013). However, human evidence remains limited, particularly with regard to whether short-term vitamin D_3_ supplementation can modify resting inflammatory status and the acute inflammatory response to repeated supramaximal exercise. Moreover, it remains unclear whether such changes are accompanied by alterations in maximal anaerobic performance.

Therefore, the aim of the present randomized double-blind trial was to evaluate the effects of 21 days of vitamin D_3_ supplementation (10,000 IU/day) on inflammatory biomarkers and maximal anaerobic performance in physically active men performing the 2 × Wingate Anaerobic Test (2xWAnT). Specifically, the study examined resting concentrations of selected inflammatory biomarkers as well as their post-exercise responses before and after the supplementation period.

## Methods

### Experimental overview

The study was designed as a randomized, double-blind, placebo-controlled parallel-group trial. The intervention consisted of 21 days of vitamin D_3_ supplementation at a dose of 10,000 IU/day.

Two weeks before the start of the intervention, participants attended an initial visit during which the study procedures were explained and eligibility was verified. One week before the experiment, all participants completed a familiarization session with the testing protocol. On the first experimental day, in the early morning hours, age, height, and body composition were assessed, participants underwent medical examination, and the 2xWAnT was performed. Venous blood samples were collected according to the experimental protocol before the test, immediately after exercise (within 3 min), and again 3 h and 24 h after the 2xWAnT. Serum vitamin D status and inflammatory biomarkers were subsequently analyzed. The same protocol was repeated after the 21-day intervention. Data collection was conducted in parallel in both groups between mid-March and mid-April, ensuring that all pre- and post-intervention assessments were completed within the same seasonal period. All laboratory analyses were performed at the Gdańsk University of Physical Education and Sport, Gdańsk, Poland.

### Participants

**Figure 1:**
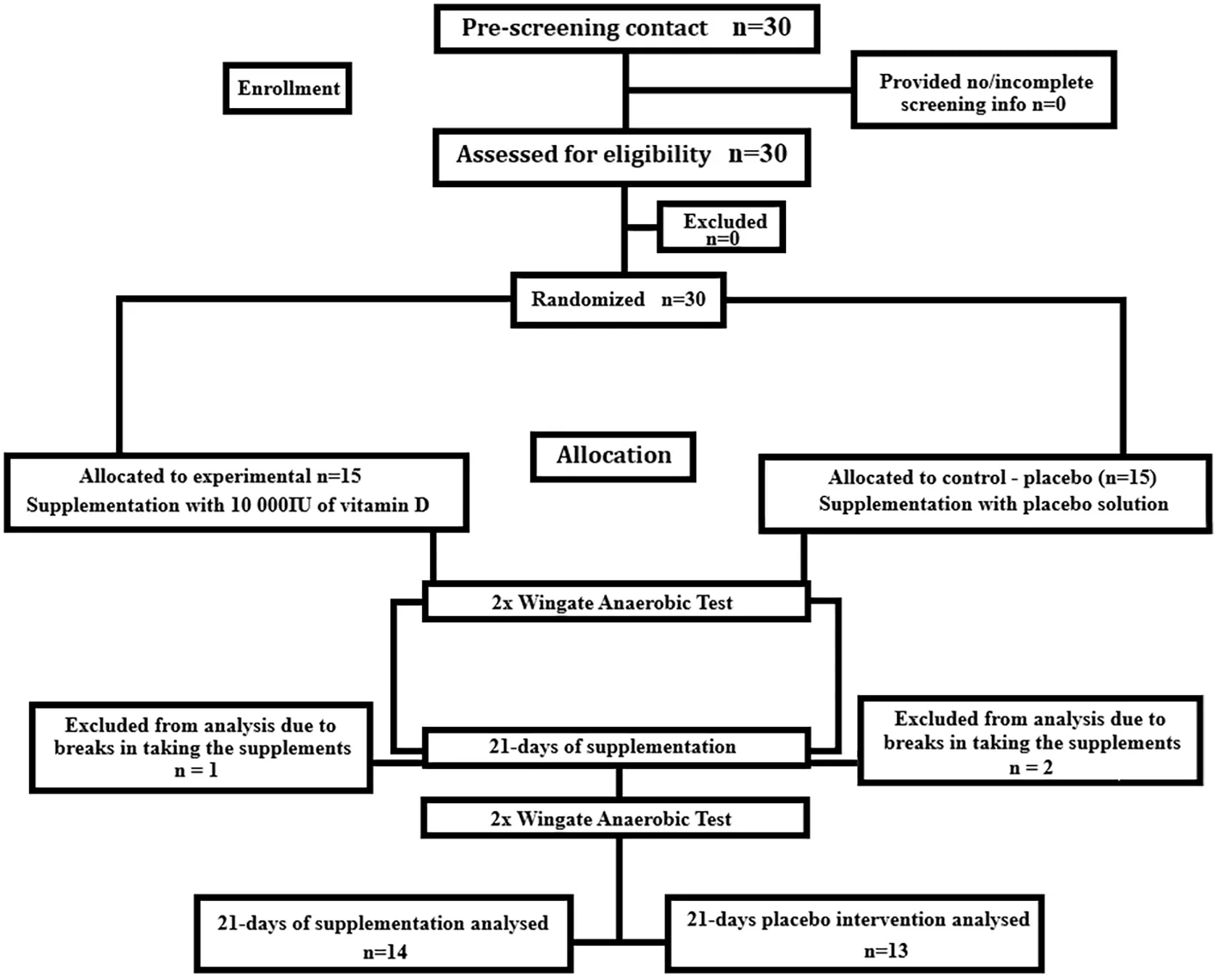
CONSORT flow diagram.

Thirty physically active men were enrolled in the study and assigned to a 21-day intervention protocol. Prior to the start of the study, participants completed an online screening questionnaire. They were then randomly assigned to either the vitamin D_3_ group (n = 15) or the placebo group (n = 15). During the intervention, one participant from the vitamin D_3_ group and two from the placebo group were excluded because of non-compliance, leaving 27 participants for the final analyses (vitamin D_3_, n = 14; placebo, n = 13; see CONSORT flow diagram, **Figure 1**).

The characteristics of both groups are presented in **Table 1**, and the eligibility criteria are summarized in **Box 1**. All participants attended a familiarization session one week before the experiment. From that time and throughout the testing period, participants were instructed to abstain from alcohol, caffeine, other stimulants, non-steroidal anti-inflammatory drugs, analgesics, and any additional dietary supplements. Participants were also instructed to maintain their habitual diet and to replicate a similar dietary pattern on each measurement day. In case of studded population, a macronutrient ratio consisted of approximately 60-70% of total daily calories from carbohydrates, 11-20% from protein, and 20-25% from fats. Such diet is analogous to the dietary guidelines for such a population of physically active people due to fact that it helps to optimize energy availability and recovery while supporting muscle function and overall health.

**Table 1:** Participant characteristics, body composition, and resting serum 25(OH)D concentrations before and after 21 days of vitamin D_3_ supplementation (mean ± SD and 95% CI).

Variables	Group	Before 21 days of vitamin D_3_ supplementation		After 21 days of vitamin D_3_ supplementation	
		Mean ± SD	95% CI	Mean ± SD	95% CI
Age (years)	Placebo	23.15 ± 3.44	21.08 - 25.23	–	–
	Vitamin D_3_	24.21 ± 4.35	21.70 - 26.73	–	–
Height (cm)	Placebo	178.15 ± 6.28	174.36 - 181.95	–	–
	Vitamin D_3_	178.36 ± 6.65	174.52 - 182.20	–	–
Body mass (kg)	Placebo	79.05 ± 8.82	73.72 - 84.37	78.66 ± 9.13	73.14 - 84.18
	Vitamin D_3_	75.01 ± 6.90	71.03 - 79.00	75.12 ± 6.87	71.15 - 79.09
BMI (kg·m^-^²)	Placebo	24.66 ± 2.64	23.07 - 26.26	24.77 ± 2.24	23.41 - 26.13
	Vitamin D_3_	23.65 ± 2.84	22.01 - 25.29	23.70 ± 2.80	22.09 - 25.31
Body fat (%)	Placebo	13.75 ± 4.45	11.07 - 16.44	14.72 ± 4.46	12.03 - 17.42
	Vitamin D_3_	13.50 ± 5.02	10.60 - 16.40	14.20 ± 5.66	10.93 - 17.47
Resting serum 25(OH)D (ng/mL)	Placebo	25.35 ± 4.13	22.85–27.84	25.47 ± 3.17	23.55–27.38
	Vitamin D_3_	27.72 ± 6.42	23.85–31.60	43.18 ± 8.12	38.27–48.08

BMI, body mass index; 25(OH)D, 25-hydroxyvitamin D; vitamin D_3_ group (n=14), placebo group (n=13).

Box 1 **Eligibility criteria.**

**Table:** 

**Inclusion criteria**	1. Individuals aged 19 to 25 years.
	2. Regular participation in recreational physical activity at least 2–3 times per week for a minimum of 45 minutes per session.
	3. Good health status during the last six months.
	4. No intake of additional medications, alcohol consumption, or smoking.
	5. No habitual smoking, regular medication use, or dietary supplement consumption.
**Exclusion criteria**	1. Individuals who are physically or mentally compromised, or who are unwilling or unable to comply with study evaluations.
	2. Comorbidities that cause severe inflammation, such as allergies, asthma, celiac disease, psoriasis, rheumatoid arthritis, type 1 diabetes, and others.
	3. Gastrointestinal issues and diseases, including irritable bowel syndrome, infectious colitis, Crohn’s disease, ulcerative colitis, gallstones, fecal incontinence, lactose intolerance, malabsorption syndromes, hepatitis, and others.
	4. Use of any antibiotics, synthetic (non-antibiotic) antimicrobial agents, or anti-inflammatory medications within six months prior to the study.

Participants’ sun exposure (one of the factors that may affect vitamin D concentration) was assessed through a questionnaire that inquired about their typical outdoor activities, the duration and frequency of sun exposure during the study period, and the use of sunscreen or protective clothing. All participants were informed that should not attend in outdoor activities that could influence vitamin D levels and, consequently, the study outcomes.

The study protocol was approved by the Bioethics Committee for Clinical Research at the Regional Medical Chamber in Gdańsk (decision no. KB-24/16) and was conducted in accordance with the Declaration of Helsinki. All participants provided written informed consent before enrollment. The trial was registered at ClinicalTrials.gov (NCT03417700).

Participants were informed about the study procedures, but they were not told their group allocation or the expected effects of supplementation in order to minimize expectation bias.

Over the preceding six months, all participants reported good health status, with no history of musculoskeletal injury, cardiovascular disease, autonomic dysfunction, or other conditions that could affect the study outcomes. All participants also reported regular recreational physical activity, including team sports, jogging, or gym-based training, performed at least 2–3 times per week for a minimum of 45 min per session.

### Vitamin D supplementation

Participants in the vitamin D_3_ group received 10,000 IU/day of vitamin D_3_ dissolved in 5 mL of vegetable oil for 21 consecutive days. The placebo group received an equivalent volume of vegetable oil matched for appearance, taste, and consistency. To preserve blinding, both preparations were provided in sealed sintered-glass bottles labeled with randomly assigned numbers, and neither the participants nor the investigators were aware of group allocation.

### Maximal anaerobic effort

Maximal anaerobic performance was assessed using two consecutive Wingate Anaerobic Tests (2xWAnT) performed on a Monark 894E cycle ergometer (Peak Bike, Sweden), according to the protocol described by Bar-Or (1987). The saddle height was adjusted individually for each participant to ensure slight knee flexion, corresponding to a final knee angle of approximately 170–175°. Before testing, participants completed a standardized 5-min warm-up on the ergometer at 60 rpm against a load of 1 W/kg. During each Wingate test, participants performed a 30-s all-out effort against a fixed braking force of 75 g/kg of total body mass. Strong verbal encouragement was provided throughout both trials to maintain maximal cadence. A 30-s passive recovery period separated the first and second Wingate tests.

The cycle ergometer was connected to a personal computer running MCE 5.1 JBA Zb software (Staniak, Poland). The following normalized performance variables were recorded during both tests: relative peak power (W/kg) and relative mean power (W/kg).

### Serum sample collection, and inflammation marker and 25(OH)D measurements

The venous blood samples were collected from the ulnar vein in 5 mL BD Vacutainer Clot Activator Tubes (Becton Dickinson and Company, NJ, USA) by a professional medical diagnostic before and after the 21-day supplementation period, according to the experimental protocol, i.e., at four time points: before the 2xWAnT, immediately after (up to 3 min after the test), 3 h and 24 h after the test. The serum was separated by centrifugation at 4,000 g for 10 min and aliquoted into 500 μL portions and frozen at −80 °C until further analysis but not longer than 6 months.

Total serum concentration of 25-hydroxyvitamin D [25(OH)D], was determined in all free from hemolysis samples and time points using the commercially available total 25OH Vitamin D ELISA kits (DRG Instruments GmbH, Germany), according to the manufacturer’s protocol. All assays were performed in duplicate and presented in **Table 2**.

**Table 2:** Two-way mixed-design ANOVA (Group × supplementation phase; 2 groups, 2 time points) for resting serum 25(OH)D and inflammatory biomarkers before and after 21 days of vitamin D_3_ supplementation.

Variable	Effect	F	Df	p	q	Effect size (ηp²)	*Post hoc*
25(OH)D	Group	22.15	1, 25	<0.001	<0.001	0.480	C < S
	Time	100.28	1, 25	<0.001	<0.001	0.807	TA > TB
	Group × Time	97.15	1, 25	<0.001	<0.001	0.802	S-TA > C-TA; within S: TA > TB
IL-1β	Group	0.06	1, 25	0.816	0.816	0.002	
	Time	21.00	1, 25	<0.001	<0.001	0.456	TB > TA
	Group × Time	7.42	1, 25	0.012	0.015	0.229	within S: TB > TA
IL-1RA	Group	0.45	1, 25	0.508	0.677	0.018	
	Time	9.23	1, 25	0.006	0.007	0.270	TB > TA
	Group × Time	0.43	1, 25	0.519	0.519	0.017	
IL-6	Group	3.15	1, 25	0.088	0.176	0.112	
	Time	3.88	1, 25	0.060	0.060	0.134	
	Group × Time	13.25	1, 25	0.001	0.005	0.346	S-TB > C-TB; within S: TB > TA
IL-10	Group	6.89	1, 25	0.015	0.058	0.216	
	Time	19.59	1, 25	<0.001	<0.001	0.439	TA > TB
	Group × Time	9.98	1, 25	0.004	0.008	0.285	S-TA > C-TA; within S: TA > TB

25(OH)D, 25-hydroxyvitamin D; IL-1β, interleukin-1 beta; IL-1RA, interleukin-1 receptor antagonist; IL-6, interleukin-6; IL-10, interleukin-10; C, control placebo group; S, vitamin D_3_ group; TB, rest before the 21-day intervention; TA, rest after the 21-day intervention. q-values represent BH-FDR-adjusted p-values. *Post hoc* comparisons are reported only for omnibus effects retained after BH-FDR correction.

Levels of the following inflammatory response and other serum markers were determined in every time point before and after 21 days of supplementation: IL-1 beta, IL-1 RA, IL-6, IL-10. The analyses were done using a MAGPIX fluorescence detection system (Luminex Corp., Austin, TX, USA) and Luminex assays (Austin, TX, USA - Luminex Human Magnetic Assay).

### Statistical analysis

Descriptive statistics are presented as mean ± standard deviation (SD). Differences in body composition and Wingate Anaerobic Test (WAnT) performance variables were evaluated using two-way mixed-design ANOVA, with group (vitamin D_3_ vs placebo) as the between-subject factor and supplementation phase (before vs after 21 days) as the within-subject factor.

Resting serum 25(OH)D concentrations and resting inflammatory biomarkers were analyzed using two-way mixed-design ANOVA with group (vitamin D_3_ vs placebo) as the between-subject factor and supplementation phase (before vs after 21 days) as the within-subject factor. To assess the acute response to exercise, separate two-way mixed-design ANOVAs were performed for the pre-supplementation and post-supplementation trials, with group (vitamin D_3_ vs placebo) as the between-subject factor and time (rest, immediately post-WAnT, 3 h post-WAnT, and 24 h post-WAnT) as the within-subject factor.

When significant ANOVA effects were detected, *post hoc* comparisons were performed using Tukey’s HSD test. To account for multiple testing, Benjamini-Hochberg false discovery rate (BH-FDR) correction was applied to families of related ANOVA effects (Benjamini and Hochberg, 1995). For inflammatory biomarkers, the correction was performed separately for the pre-supplementation and post-supplementation analyses. Statistical significance for ANOVA effects was accepted at q < 0.05, whereas significance for Tukey’s HSD *post hoc* comparisons was accepted at p < 0.05.

Effect sizes are reported as partial eta-squared (ηp²), with thresholds of 0.01, 0.06, and 0.14 interpreted as small, moderate, and large effects, respectively. *A priori* power analysis was performed using G*Power version 3.1.9.2, indicating that a minimum sample size of 24 participants was required to detect a medium interaction effect with 80% power. All graphical representations were created using GraphPad Prism 6.0 (GraphPad Software, MA, USA), and all statistical analyses were performed using Statistica 12 (StatSoft, Tulsa, OK, USA).

## Results

Results are reported with BH-FDR-adjusted q-values; p-values accompanying percentage changes refer to *post hoc* comparisons.

### Wingate anaerobic test outcomes: effect of 21-day vitamin D_3_ supplementation

**Table 3** presents Wingate outcomes before and after the 21-day vitamin D_3_ supplementation. No differential effect of vitamin D_3_ versus placebo was observed for maximal anaerobic performance.

**Table 3:** Double 30-s Wingate anaerobic test (WAnT) outcomes before and after 21 days of vitamin D_3_ supplementation (mean ± SD and 95% CI).

Variable	Unit	Group	Before 21 days of vitamin D_3_ supplementation		After 21 days of vitamin D_3_ supplementation	
			Mean ± SD	95% CI	Mean ± SD	95% CI
Relative mean power (1st WAnT)	W·kg^-^¹	Placebo	7.62 ± 0.81	7.13–8.11	7.58 ± 0.79	7.10–8.06
		Vitamin D_3_	7.85 ± 0.43	7.60–8.10	7.77 ± 0.35	7.57–7.97
Relative mean power (2nd WAnT)	W·kg^-^¹	Placebo	5.19 ± 0.71	4.76–5.62	5.27 ± 0.91	4.72–5.82
		Vitamin D_3_	4.93 ± 1.05	4.32–5.53	5.11 ± 0.61	4.76–5.46
Relative peak power (1st WAnT)	W·kg^-^¹	Placebo	10.11 ± 1.29	9.33–10.88	9.52 ± 1.67	8.51–10.53
		Vitamin D_3_	10.26 ± 0.86	9.77–10.76	10.35 ± 0.80	9.89–10.81
Relative peak power (2nd WAnT)	W·kg^-^¹	Placebo	7.17 ± 0.97	6.58–7.75	6.96 ± 0.90	6.42–7.50
		Vitamin D_3_	7.07 ± 0.88	6.56–7.58	7.21 ± 0.52	6.91–7.51

CI, confidence interval; WAnT, Wingate anaerobic test, vitamin D_3_ (n=14), placebo (n=13).

### Resting serum 25(OH)D and inflammatory biomarkers: effect of 21-day vitamin D_3_ supplementation

Resting serum 25(OH)D concentrations before and after the 21-day intervention are presented in **Table 1**. Before the intervention, 25(OH)D concentrations did not differ between the placebo and vitamin D_3_ groups (p = 0.724). **Table 2** summarizes the ANOVA results for resting serum 25(OH)D and inflammatory biomarkers before and after the 21-day intervention. After 21 days, resting 25(OH)D increased in the vitamin D_3_ group by +55.7% (p < 0.001), whereas no significant change was observed in placebo (+0.5%, p = 1.000). Consequently, after the intervention, resting 25(OH)D concentrations were higher in the vitamin D_3_ group than in placebo (p < 0.001).

For inflammatory biomarkers, significant Group × supplementation phase interactions were observed for IL-1β, IL-6, and IL-10, whereas no interaction was found for IL-1RA. In the vitamin D_3_ group, resting IL-1β decreased by −15.8% after supplementation (p < 0.001), while the change in placebo was not significant (−4.2%, p = 0.578). Resting IL-6 was higher in the vitamin D_3_ group than in placebo at baseline (+18.9%, p = 0.047), but this between-group difference was no longer present after the intervention (p = 0.916). Within the vitamin D_3_ group, IL-6 decreased by −9.4% after supplementation (p = 0.002), whereas no significant change was observed in placebo (+3.3%, p = 0.657). Resting IL-10 increased in the vitamin D_3_ group by +17.2% (p < 0.001), with no significant change in placebo (+3.6%, p = 0.815). After the intervention, IL-10 concentrations were higher in the vitamin D_3_ group than in placebo (+42.3%, p = 0.016). For IL-1RA, no Group × supplementation phase interaction was observed (p = 0.519), although a supplementation phase effect indicated lower resting concentrations after 21 days irrespective of group (−11.8%, p = 0.006).

### Serum 25(OH)D response to the Wingate anaerobic test before and after 21 days of vitamin D_3_ supplementation

**Figure 2:**
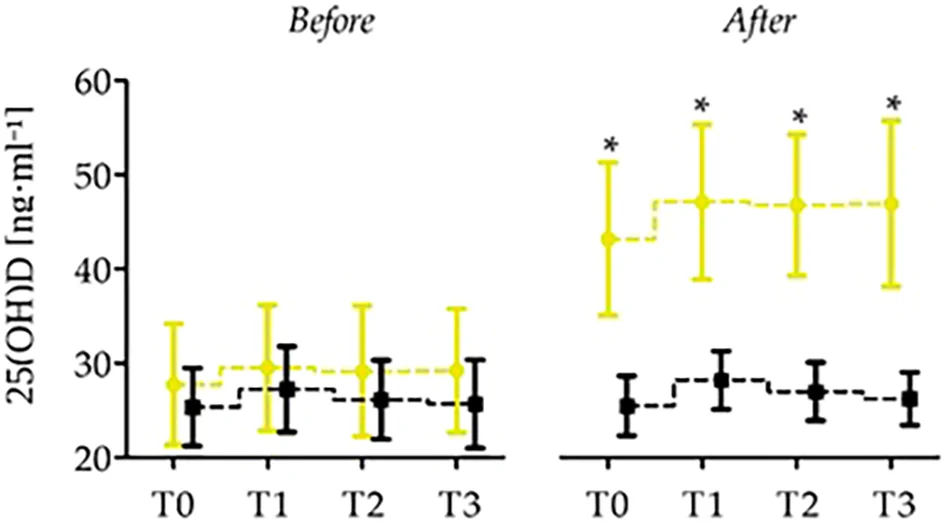
Changes in 25-hydroxyvitamin D (25(OH)D) in response to the Wingate anaerobic test (WAnT) before and after 21 days of vitamin D_3_ supplementation (mean ± SD) (yellow – Vit D, black – control). T0, resting; T1, immediately post-WAnT; T2, 3 h post-WAnT; T3, 24 h post-WAnT; * significant between-group difference at the corresponding time point (p < 0.01).

**Table 4** summarizes the ANOVA results for changes in 25(OH)D in response to the WAnT before and after the 21-day intervention. Before supplementation, 25(OH)D increased by +6.9% immediately post-WAnT compared with resting values (p < 0.01) and remained elevated at 3 h and 24 h post-WAnT (both p < 0.01), irrespective of group. After 21 days, a comparable acute response pattern was observed; however, 25(OH)D concentrations were consistently higher in the vitamin D group than in placebo at rest (+69.5%), immediately post-WAnT (+67.0%), 3 h post-WAnT (+73.3%), and 24 h post-WAnT (+78.9%) (all p < 0.01), corresponding to an average between-group difference of approximately +72.2% across all four time points. (**Figure 2**).

**Table 4:** Two-way repeated-measures ANOVA (Group × Time; 2 groups, 4 time points) for serum 25(OH)D concentrations in response to the Wingate anaerobic test (WAnT), assessed before and after 21 days of vitamin D_3_ supplementation.

Variable	Intervention	Effect	F	Df	p	q	Effect size (ηp²)	*Post hoc*
25(OH)D	Before	Group	1.6763	1,24	0.208	0.208	0.06	
		Time	10.7771	3,72	<0.001	<0.001	0.31	T1, T2, T3 > T0
		Group × Time	1.7117	3,72	0.172	0.208	0.06	
	After	Group	65.3662	1,24	<0.001	<0.001	0.73	C < S
		Time	39.7121	3,72	<0.001	<0.001	0.62	T1, T2, T3 > T0
		Group × Time	7.8881	3,72	<0.001	<0.001	0.25	C-T0-T3 < S-T0-T3

25(OH)D, 25-hydroxyvitamin D; C, control (placebo); S, vitamin D_3_; T0, rest; T1, immediately post-WAnT; T2, 3 h post-WAnT; T3, 24 h post-WAnT. q-values represent BH-FDR-adjusted p-values. *Post hoc* comparisons are reported only for omnibus effects retained after BH-FDR correction.

### Inflammatory biomarkers: response - the Wingate anaerobic test before and after 21 days of vitamin D_3_ supplementation

Inflammatory biomarker concentrations were assessed at rest, immediately post-WAnT, 3 h post-WAnT, and 24 h post-WAnT. ANOVA results are presented in **Table 5**, and only *post hoc* comparisons corresponding to effects retained after BH-FDR adjustment are described below.

**Table 5:** Two-way repeated-measures ANOVA (Group × Time; 2 groups, 4 time points) for inflammatory biomarkers in response to the Wingate anaerobic test (WAnT), assessed before and after 21 days of vitamin D_3_ supplementation.

Variable	Inter-vention	Effect	F	Df	p	q	Effect size (ηp²)	*Post hoc*
IL-1 beta	Before	Group	1.51	1, 25	0.23	0.309	0.06	
		Time	34.25	3, 75	< 0.001	<0.001	0.58	T1 > T0, T2, T3; T2 > T0, T3
		Group × Time	2.22	3, 75	0.093	0.186	0.08	
	After	Group	33.78	1, 25	< 0.001	<0.001	0.57	S < C
		Time	19.68	3, 75	< 0.001	<0.001	0.44	T1 > T0, T2, T3; T2 > T3
		Group × Time	7.86	3, 75	< 0.001	<0.001	0.24	C-T1 > S-T1; C-T2 > S-T2; C-T3 > S-T3; within C: T1, T2 > T0; T1 > T3; within S: T0, T1 > T3
IL-1 RA	Before	Group	0.07	1, 25	0.79	0.787	0.00	
		Time	28.53	3, 75	< 0.001	<0.001	0.53	T1, T2 > T0; T3 > T0; T1, T2 > T3
		Group × Time	2.49	3, 75	0.067	0.186	0.09	
	After	Group	13.45	1, 25	0.001	0.002	0.35	S < C
		Time	23.80	3, 75	< 0.001	<0.001	0.49	T1, T2 > T0; T3 > T0; T1, T2 > T3
		Group × Time	8.74	3, 75	< 0.001	<0.001	0.26	C-T1 > S-T1; C-T2 > S-T2; C-T3 > S-T3; within C: T1, T2, T3 > T0; T2 > T3
IL-6	Before	Group	7.30	1, 25	0.019	0.082	0.20	
		Time	26.10	3, 75	< 0.001	<0.001	0.51	T1, T2 > T0, T3
		Group × Time	0.71	3, 75	0.551	0.551	0.02	
	After	Group	0.09	1, 25	0.764	0.764	0.01	
		Time	10.37	3, 75	< 0.001	<0.001	0.29	T1, T2 > T0, T3
		Group × Time	1.54	3, 75	0.210	0.210	0.05	
IL-10	Before	Group	4.63	1, 25	0.048	0.112	0.16	
		Time	14.77	3, 75	< 0.001*	<0.001	0.37	T1 > T0, T2, T3
		Group × Time	1.13	3, 75	0.34	0.458	0.04	
	After	Group	43.31	1, 25	< 0.001	<0.001	0.63	C < S
		Time	17.72	3, 75	< 0.001	<0.001	0.41	T1 > T0, T2, T3; T2 > T3
		Group × Time	4.08	3, 75	0.010	0.013	0.14	S-T0 > C-T0; S-T1 > C-T1; S-T2 > C-T2; S-T3 > C-T3; within S: T1, T2 > T0, T3

IL-1β, interleukin-1 beta; IL-1RA, interleukin-1 receptor antagonist; IL-6, interleukin-6; IL-10, interleukin-10; C, control (placebo); S, vitamin D_3_; T0, rest; T1, immediately post-WAnT; T2, 3 h post-WAnT; T3, 24 h post-WAnT. q-values represent BH-FDR-adjusted p-values. *Post hoc* comparisons are reported only for omnibus effects retained after BH-FDR correction across the four inflammatory biomarkers within each intervention phase and ANOVA effect family.

Before supplementation, all biomarkers showed a time-dependent response to the WAnT. Compared with resting values, IL-1β increased immediately post-WAnT by +41.6% (p < 0.001) and remained elevated at 3 h post-WAnT by +19.3% (p = 0.001). IL-1RA increased immediately post-WAnT by +47.7% (p < 0.001), remained elevated at 3 h post-WAnT by +55.7% (p < 0.001), and was still above resting values at 24 h post-WAnT by +21.6% (p = 0.010). IL-6 increased immediately post-WAnT by +42.1% (p < 0.001) and remained elevated at 3 h post-WAnT by +39.3% (p < 0.001). IL-10 showed a transient increase immediately post-WAnT by +21.5% (p < 0.001), with no significant differences between rest and the later recovery time points.

**Figure 3:**
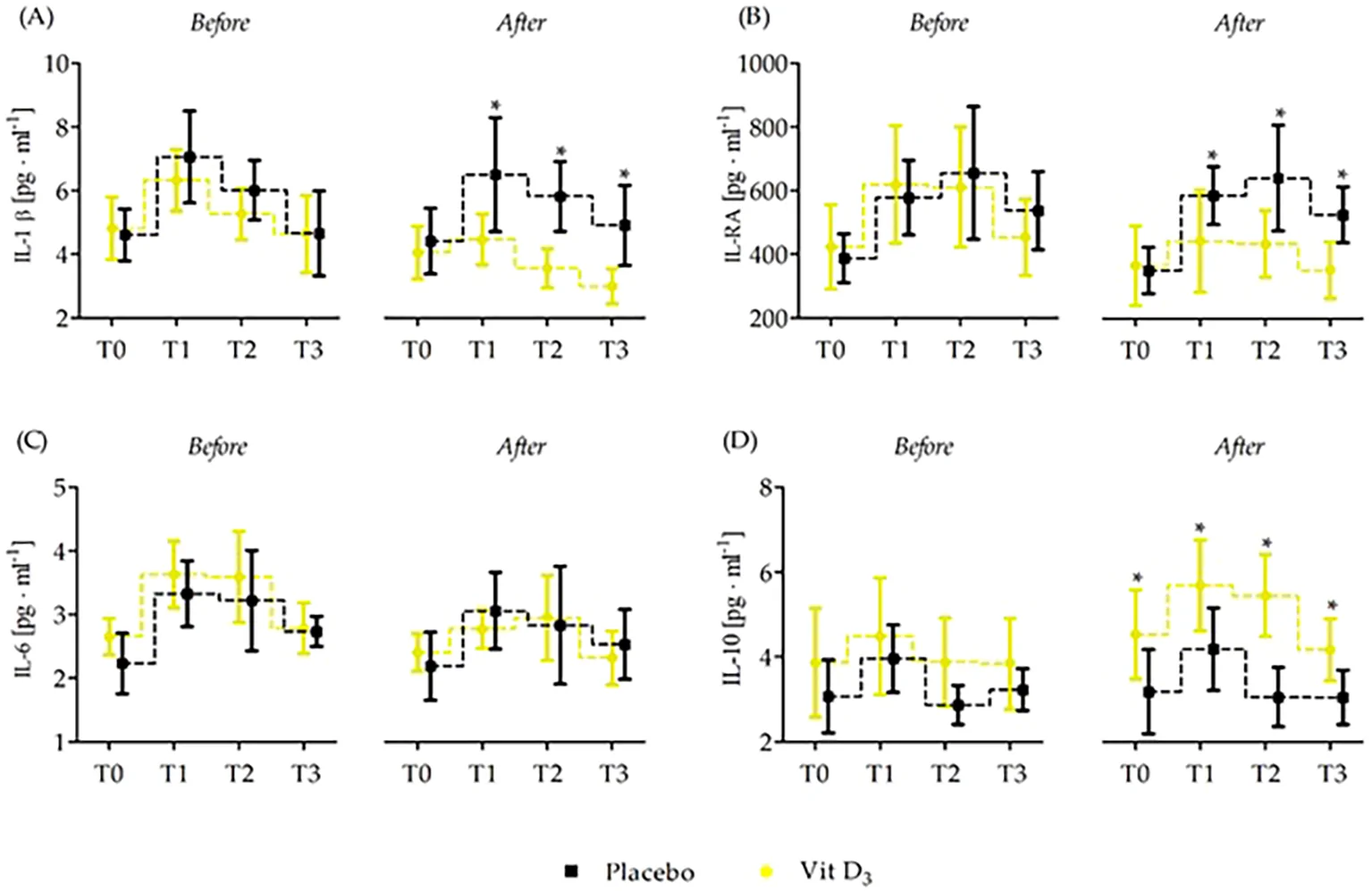
Changes in inflammatory biomarkers in response to the Wingate anaerobic test (WAnT) before and after 21 days of vitamin D_3_ supplementation (mean ± SD). Abbreviations/Symbols: IL-1β, interleukin-1 beta **(A)**; IL-1RA, interleukin-1 receptor antagonist **(B)**; IL-6, interleukin-6 **(C)**; IL-10, interleukin-10 **(D)**; T0, resting; T1, immediately post-WAnT; T2, 3 h post-WAnT; T3, 24 h post-WAnT; * significant between-group difference at the p < 0.05.

After 21 days, IL-1β showed a blunted post-exercise response in the vitamin D_3_ group. In placebo, IL-1β increased from rest to immediately post-WAnT by +47.3% (p < 0.001), whereas the corresponding change in the vitamin D_3_ group was small and non-significant (+10.2%, p = 0.897). Placebo values were higher than vitamin D_3_ immediately post-WAnT by +45.6% (p < 0.001), at 3 h post-WAnT by +63.2% (p < 0.001), and at 24 h post-WAnT by +82.3% (p < 0.001) (**Figure 3**).

A similar pattern was observed for IL-1RA. In the placebo group, IL-1RA increased from rest to immediately post-WAnT by +67.2% (p < 0.001), whereas the increase in the vitamin D_3_ group was smaller and non-significant (+21.2%, p = 0.273). No between-group difference was observed at rest (p = 1.000), but placebo values were higher than vitamin D_3_ immediately post-WAnT by +32.2% (p = 0.046), at 3 h post-WAnT by +47.7% (p = 0.001), and at 24 h post-WAnT by +49.2% (p = 0.007). Within the placebo group, IL-1RA remained elevated above rest at 3 h post-WAnT (+82.9%, p < 0.001) and 24 h post-WAnT (+50.0%, p < 0.001).

For IL-10, concentrations were higher in the vitamin D_3_ group than in placebo at all time points after supplementation, including rest. The between-group differences were +42.3% at rest (p = 0.004), +36.0% immediately post-WAnT (p < 0.001), +78.0% at 3 h post-WAnT (p < 0.001), and +36.8% at 24 h post-WAnT (p = 0.030). In the placebo group, IL-10 declined from immediately post-WAnT to 3 h post-WAnT by −26.9% (p = 0.003), with no difference between 3 h post-WAnT and rest (p = 1.000). In the vitamin D_3_ group, the decrease from immediately post-WAnT to 3 h post-WAnT was small and non-significant (−4.2%, p = 0.986). Within the vitamin D_3_ group, IL-10 remained higher immediately post-WAnT than at rest (+25.4%, p = 0.001) and higher at 3 h post-WAnT than at rest (+20.1%, p = 0.024).

For IL-6, concentrations were higher immediately post-WAnT than at rest by +26.5% (p < 0.001) and remained elevated at 3 h post-WAnT by +25.7% (p < 0.001), with no significant between-group differences at any time point.

## Discussion

The present study examined the effects of 21 days of vitamin D_3_ supplementation on serum 25(OH)D concentrations, maximal anaerobic performance, and inflammatory responses in physically active men. Our findings revealed a significant increase in serum 25(OH)D levels (+55.7%) in the vitamin D_3_ group, consistent with previous research that has documented the efficacy of vitamin D_3_ supplementation in elevating serum vitamin D levels (Han et al., 2019; Mieszkowski et al., 2021c; Sist et al., 2023). This increase is particularly noteworthy as adequate vitamin D levels are essential for various physiological processes, including bone homeostasis (Mieszkowski et al., 2021a), and metabolism (Girgis et al., 2013), muscle function, and metabolic regulation (Mieszkowski et al., 2021b; Wicherts et al., 2007; Girgis et al., 2013).

Despite the marked increase in serum 25(OH)D concentrations, we observed no improvement in Wingate Anaerobic Test (WAnT) performance. This finding suggests that while vitamin D_3_ supplementation enhances vitamin D status, it does not directly translate to improvements in maximal anaerobic performance in physically active men. Previous studies have reported similar findings, indicating that enhancements in vitamin D levels do not necessarily correlate with increased maximal strength, power, or aerobic performance (Todd et al., 2017; Książek et al., 2019; Sist et al., 2023). This may be attributed to the fact that our participants were not selected based on significant vitamin D deficiency. It has been suggested that individuals with low baseline vitamin D levels may experience more pronounced performance benefits from supplementation, while those with adequate levels may not see any substantial changes (Ogan and Pritchett, 2013).

The relationship between vitamin D status and physical performance is complex and multifaceted. Several studies have identified a threshold effect, indicating that improvements in neuromuscular performance are most significant when serum 25(OH)D levels are between 40 and 50 ng/mL (Bischoff-Ferrari et al., 2004; Wicherts et al., 2007). While some longitudinal studies have shown associations, others have not (Książek et al., 2016; Todd et al., 2017), raising the possibility of reverse causation (Bartali et al., 2008). Our findings suggest that while the vitamin D_3_ supplementation successfully elevated serum levels, it may not have reached the threshold necessary to elicit performance enhancements in our cohort. In terms of inflammatory responses, our study identified significant alterations in resting inflammatory biomarkers following vitamin D_3_ supplementation.

The vitamin D_3_ group exhibited notable reductions in IL-1β (−15.8%) and IL-6 (−9.4%), alongside an increase in IL-10 (+17.2%). These changes indicate a favorable anti-inflammatory response, suggesting that vitamin D_3_ may play a critical role in modulating inflammation, which is particularly relevant for recovery in physically active individuals. Previous research has demonstrated that cholecalciferol can reduce circulating levels of pro-inflammatory markers, although the effects have not been uniform across different populations and studies (Grossmann et al., 2012; Chandler et al., 2014; Salekzamani et al., 2017; Rodriguez et al., 2018).

Moreover, the post-exercise inflammatory response also differed significantly between the vitamin D_3_ and placebo groups. The vitamin D_3_ group showed an attenuated IL-1β response following the 2xWAnT, lower IL-1RA concentrations during the post-exercise period, and higher IL-10 concentrations throughout recovery. This aligns in part with studies by Mieszkowski et al (2021a), which suggest that vitamin D_3_ supplementation may facilitate a more favorable recovery profile by modulating inflammatory responses after intense exercise, potentially leading to improved performance in subsequent training sessions (Thomas and Larson-Meyer, 2013; Koundourakis et al., 2014; Fitzgerald et al., 2015).

However, it is important to note that not all studies have consistently demonstrated a reduction in post-exercise IL-6 levels after vitamin D_3_ supplementation, mirroring the lack of between-group differences in our findings. This variability highlights the need for further research to elucidate the complexities of the inflammatory response to vitamin D supplementation in diverse populations and exercise modalities.

The observed attenuation of post-exercise IL-1β and IL-1RA responses, together with higher IL-10 concentrations, may reflect an enhanced recovery profile, which could facilitate improved performance in subsequent training sessions (Thomas and Larson-Meyer, 2013; Koundourakis et al., 2014; Fitzgerald et al., 2015). The differential inflammatory response is particularly relevant in the context of exercise recovery, where excessive inflammation can hinder adaptation (Rojano-Ortega and Berral-de la Rosa, 2023). Like it was shown in work by Rojano-Ortega and Berral-de la Rosa (2023), such proposed supplementation strategy with the use of vitamin D supplementation for periods of more than 1 week with a minimum dose of 2000 IU/day appears to be an efficacious strategy for attenuating muscle damage and inflammation after exercise.

## Limitations and future directions

While the findings of this study offer valuable insights into the effects of vitamin D_3_ supplementation, several limitations warrant consideration. The sample size, although appropriate for detecting medium effect sizes, may not capture the full variability in responses, limiting generalizability. Macronutrient and antioxidant intake, as well as training load, were not objectively recorded; therefore, unreported variation in these factors may have contributed to variability in recovery and inflammatory biomarkers. Recovery was assessed only through inflammatory biomarkers, without functional recovery measures. Therefore, the present findings are limited to biochemical aspects of the post-exercise response and do not demonstrate improved functional recovery following vitamin D_3_ supplementation. Additionally, because the 2xWAnT is a specific acute supramaximal anaerobic exercise protocol, the observed inflammatory response reflects the physiological demands of this particular exercise model. The short duration of supplementation may not have been sufficient to observe longer-term effects on performance. Future research should include standardized dietary records and training-load monitoring, explore longer supplementation durations, and incorporate varied performance measures, such as endurance and strength assessments, to gain a more comprehensive understanding of vitamin D’s impact. Investigating the underlying mechanisms, such as the role of vitamin D in muscle cell signaling and immune response, could provide further insights into its influence on athletic performance and recovery. Moreover, because the response to vitamin D_3_ supplementation may depend on baseline 25(OH)D status, future studies should stratify participants according to vitamin D status and determine whether individuals with insufficient concentrations derive greater performance- or recovery-related benefits from supplementation. This would be consistent with previous suggestions that physically active populations may require adequate vitamin D availability to support musculoskeletal and metabolic processes relevant to performance Ogan and Pritchett (2013).

## Conclusion

In conclusion, 21 days of vitamin D_3_ supplementation significantly increased serum 25(OH)D concentrations but did not improve anaerobic performance. Supplementation was associated with selected changes in resting and post-WAnT inflammatory biomarkers, suggesting modulation of the biochemical inflammatory response to repeated supramaximal anaerobic exercise. However, because functional recovery outcomes were not assessed, these findings do not demonstrate improved functional recovery. Further studies including functional recovery measures and different exercise models are needed to determine the practical relevance of these effects.

## What are the findings?

- 21-day 10–000 IU vitamin D supplementation increased total serum 25(OH)D concentrations.
- The 2xWAnT induced an acute increase in serum 25(OH)D concentrations
- 21-day supplementation with 10,000 IU of vitamin D lowered resting IL-1β and IL-6 and increased resting IL-10. After the 2xWAnT, it was associated with an attenuated IL-1β response, lower IL-1RA concentrations during the post-exercise period, and higher IL-10 concentrations, whereas the post-exercise IL-6 time course did not differ between groups.

## How might it impact on clinical practice in the future?

- Targeted supplementation: The demonstrated increase in serum 25(OH)D levels following 21 days of 10,000 IU vitamin D supplementation highlights the importance of tailored vitamin D administration in physically active populations, particularly those with low 25(OH)D baseline levels.
- Biochemical monitoring of post-exercise responses: The observed modulation of inflammatory biomarkers, including lower resting IL-1β and IL-6, higher resting IL-10, an attenuated post-exercise IL-1β response, lower IL-1RA concentrations during the post-exercise period, and higher IL-10 concentrations, suggests that vitamin D supplementation may support regulation of exercise-induced inflammation. However, direct effects on recovery time and performance require further confirmation.
- Preventive health strategy: Given the positive effects on inflammatory markers, vitamin D supplementation may be considered a preventive measure against exercise-induced inflammation, potentially benefiting individuals at risk of inflammatory conditions.
- Further research and guidelines: These results could prompt further research into the optimal dosages and duration of vitamin D supplementation for various populations, potentially leading to updated clinical guidelines regarding vitamin D intake for improving health and recovery outcomes especially in physical active healthy populations.

## Data Availability

The raw data supporting the conclusions of this article will be made available by the authors, without undue reservation.

## References

[B1] Bar-Or O. (1987). The Wingate anaerobic test. An update on methodology, reliability and validity. Sports Med. 4, 381–394. 10.2165/00007256-198704060-00001 3324256

[B2] Bartali B. Frongillo E. A. Guralnik J. M. Stipanuk M. H. Allore H. G. Cherubini A. . (2008). Serum micronutrient concentrations and decline in physical function among older persons. Jama 299, 308–315. doi: 10.1001/jama.299.3.308 18212315 PMC2645790

[B3] Benjamini Y. Hochberg Y. (1995). Controlling the false discovery rate: a practical and powerful approach to multiple testing. J. R. Stat. Soc. Ser. B. Stat. Method. 57, 289–300. doi: 10.1111/j.2517-6161.1995.tb02031.x40046247

[B4] Bischoff-Ferrari H. A. Dietrich T. Orav E. J. Hu F. B. Zhang Y. Karlson E. W. . (2004). Higher 25-hydroxyvitamin D concentrations are associated with better lower-extremity function in both active and inactive persons aged > or =60 y. Am. J. Clin. Nutr. 80, 752–758. doi: 10.1093/ajcn/80.3.752 15321818

[B5] Chandler P. D. Scott J. B. Drake B. F. Ng K. Manson J. E. Rifai N. . (2014). Impact of vitamin D supplementation on inflammatory markers in African Americans: results of a four-arm, randomized, placebo-controlled trial. Cancer Prev. Res. 7, 218–225. doi: 10.1158/1940-6207.capr-13-0338-t 24327720 PMC4038929

[B6] Choi M. Park H. Cho S. Lee M. (2013). Vitamin D3 supplementation modulates inflammatory responses from the muscle damage induced by high-intensity exercise in SD rats. Cytokine 63, 27–35. doi: 10.1016/j.cyto.2013.03.018 23669253

[B7] Fitzgerald J. S. Peterson B. J. Warpeha J. M. Johnson S. C. Ingraham S. J. (2015). Association between vitamin D status and maximal-intensity exercise performance in junior and collegiate hockey players. J. Strength Cond Res. 29, 2513–2521. doi: 10.1519/jsc.0000000000000887 26313575

[B8] Girgis C. M. Clifton-Bligh R. J. Hamrick M. W. Holick M. F. Gunton J. E. (2013). The roles of vitamin D in skeletal muscle: form, function, and metabolism. Endocr. Rev. 34, 33–83. doi: 10.1210/er.2012-1012 23169676

[B9] Grossmann R. E. Zughaier S. M. Liu S. Lyles R. H. Tangpricha V. (2012). Impact of vitamin D supplementation on markers of inflammation in adults with cystic fibrosis hospitalized for a pulmonary exacerbation. Eur. J. Clin. Nutr. 66, 1072–1074. doi: 10.1038/ejcn.2012.82 22805498 PMC3638806

[B10] Han Q. Li X. Tan Q. Shao J. Yi M. (2019). Effects of vitamin D3 supplementation on serum 25(OH)D concentration and strength in athletes: a systematic review and meta-analysis of randomized controlled trials. J. Int. Soc Sports Nutr. 16, 019–0323. doi: 10.1186/s12970-019-0323-6 31771586 PMC6878631

[B11] Jablonski K. L. Chonchol M. Pierce G. L. Walker A. E. Seals D. R. (2011). 25-Hydroxyvitamin D deficiency is associated with inflammation-linked vascular endothelial dysfunction in middle-aged and older adults. Hypertension 57, 63–69. doi: 10.1161/hypertensionaha.110.160929 21115878 PMC3020150

[B12] Koundourakis N. E. Androulakis N. E. Malliaraki N. Margioris A. N. (2014). Vitamin D and exercise performance in professional soccer players. PloS One 9 (7). doi: 10.1371/journal.pone.0101659 24992690 PMC4081585

[B13] Książek A. Zagrodna A. Dziubek W. Pietraszewski B. Ochmann B. Słowińska-Lisowska M. (2016). 25(OH)D(3) levels relative to muscle strength and maximum oxygen uptake in athletes. J. Hum. Kinet. 50, 71–77. 10.1515/hukin-2015-0144 PMC5260642 28149343

[B14] Książek A. Zagrodna A. Słowińska-Lisowska M. (2019). Vitamin D, skeletal muscle function and athletic performance in athletes-a narrative review. Nutrients 11 (8). 10.3390/nu11081800 PMC6722905 31382666

[B15] Matilainen J. M. Räsänen A. Gynther P. Väisänen S. (2010). The genes encoding cytokines IL-2, IL-10 and IL-12B are primary 1alpha,25(OH)2D3 target genes. J. Steroid Biochem. Mol. Biol. 121, 142–145. doi: 10.1016/j.jsbmb.2010.03.020 20236616

[B16] McFadden B. A. Cintineo H. P. Chandler A. J. Arent S. M. (2022). “ Chapter 13 - Physical activity and inflammation: acute and chronic considerations,” in Diet, Inflammation, and Health. Eds. Hébert J. R. Hofseth L. J. ( Academic Press), 665–691.

[B17] Mieszkowski J. Borkowska A. Stankiewicz B. Kochanowicz A. Niespodziński B. Surmiak M. . (2021a). Single high-dose vitamin D supplementation as an approach for reducing ultramarathon-induced inflammation: a double-blind randomized controlled trial. Nutrients 13 (4). doi: 10.3390/nu13041280 33924645 PMC8069287

[B18] Mieszkowski J. Kochanowicz A. Piskorska E. Niespodziński B. Siódmiak J. Buśko K. . (2021b). Serum levels of bone formation and resorption markers in relation to vitamin D status in professional gymnastics and physically active men during upper and lower body high-intensity exercise. J. Int. Soc Sports Nutr. 18, 021–00430. doi: 10.1186/s12970-021-00430-8 33849553 PMC8045337

[B19] Mieszkowski J. Stankiewicz B. E. Kochanowicz A. Niespodziński B. Borkowska A. E. Sikorska K. . (2021c). Remote ischemic preconditioning reduces marathon-induced oxidative stress and decreases liver and heart injury markers in the serum. Front. Physiol. 12. doi: 10.3389/fphys.2021.731889 34552508 PMC8450527

[B20] Nemkov T. Stauffer E. Cendali F. Stephenson D. Nader E. Robert M. . (2025). “ Long-distance trail running induces inflammatory-associated protein, lipid, and purine oxidation in red blood cells,” in Biorxiv, vol. 19. , 648006. Preprint. 10.1016/j.brci.2026.100055 PMC13249458 42273167

[B21] Ogan D. Pritchett K. (2013). Vitamin D and the athlete: risks, recommendations, and benefits. Nutrients 5, 1856–1868. doi: 10.3390/nu5061856 23760056 PMC3725481

[B22] Rodriguez A. J. Mousa A. Ebeling P. R. Scott D. de Courten B. (2018). Effects of vitamin D supplementation on inflammatory markers in heart failure: a systematic review and meta-analysis of randomized controlled trials. Sci. Rep. 8, 018–19708. doi: 10.1038/s41598-018-19708-0 29348609 PMC5773527

[B23] Rojano-Ortega D. Berral-de la Rosa F. J. (2023). Effects of vitamin D supplementation on muscle function and recovery after exercise-induced muscle damage: a systematic review. J. Hum. Nutr. Diet. 36, 1068–1078. doi: 10.1111/jhn.13084 36149089

[B24] Salekzamani S. Bavil A. S. Mehralizadeh H. Jafarabadi M. A. Ghezel A. Gargari B. P. (2017). The effects of vitamin D supplementation on proatherogenic inflammatory markers and carotid intima media thickness in subjects with metabolic syndrome: a randomized double-blind placebo-controlled clinical trial. Endocrine 57, 51–59. doi: 10.1007/s12020-017-1317-2 28509078

[B25] Sist M. Zou L. Galloway S. D. R. Rodriguez-Sanchez N. (2023). Effects of vitamin D supplementation on maximal strength and power in athletes: a systematic review and meta-analysis of randomized controlled trials. Front. Nutr. 10, 1163313. doi: 10.3389/fnut.2023.1163313 37841405 PMC10570740

[B26] Stankiewicz B. Cieślicka M. Mieszkowski J. Kochanowicz A. Niespodziński B. Szwarc A. . (2023). Effect of supplementation with black chokeberry (Aronia melanocarpa) extract on inflammatory status and selected markers of iron metabolism in young football players: a randomized double-blind trial. Nutrients 15 (4). doi: 10.3390/nu15040975 36839333 PMC9965193

[B27] Thomas J. J. Larson-Meyer D. E. (2013). “ Vitamin D and exercise performance,” in Endocrinology of Physical Activity and Sport: Second Edition. Eds. Constantini N. Hackney A. C. ( Humana Press, Totowa, NJ), 339–362.

[B28] Todd J. J. McSorley E. M. Pourshahidi L. K. Madigan S. M. Laird E. Healy M. . (2017). Vitamin D(3) supplementation using an oral spray solution resolves deficiency but has no effect on VO(2) max in Gaelic footballers: results from a randomised, double-blind, placebo-controlled trial. Eur. J. Nutr. 56, 1577–1587. doi: 10.1007/s00394-016-1202-4 27015912 PMC5486642

[B29] Wicherts I. S. van Schoor N. M. Boeke A. J. Visser M. Deeg D. J. Smit J. . (2007). Vitamin D status predicts physical performance and its decline in older persons. J. Clin. Endocrinol. Metab. 92, 2058–2065. doi: 10.1210/jc.2006-1525 17341569

